# Thyroid Health and Selenium: The Critical Role of Adequate Intake from Fetal Development to Adolescence

**DOI:** 10.3390/nu17142362

**Published:** 2025-07-18

**Authors:** Valeria Calcaterra, Hellas Cena, Ilaria Anna Maria Scavone, Ilaria Zambon, Silvia Taranto, Cecilia Ricciardi Rizzo, Chiara Ferrara, Marianna Diotti, Gianvincenzo Zuccotti

**Affiliations:** 1 Department of Internal Medicine and Therapeutics, University of Pavia, 27100 Pavia, Italy; valeria.calcaterra@unipv.it; 2 Pediatric Department, Buzzi Children’s Hospital, 20154 Milan, Italy; ilaria.scavone@unimi.it (I.A.M.S.); silvia.taranto@unimi.it (S.T.); marianna.diotti@unimi.it (M.D.); gianvincenzo.zuccotti@unimi.it (G.Z.); 3 Laboratory of Dietetics and Clinical Nutrition, Department of Public Health, Experimental and Forensic Medicine, University of Pavia, 27100 Pavia, Italy; ilaria.zambon01@universitadipavia.it (I.Z.); cecilia.ricciardirizzo@unipv.it (C.R.R.); chiara.ferrara01@unipv.it (C.F.); 4 Clinical Nutrition Unit, General Medicine, ICS Maugeri IRCCS, 27100 Pavia, Italy; 5 Department of Biomedical and Clinical Science, University of Milano, 20157 Milan, Italy

**Keywords:** selenium, thyroid, pediatrics, children, pregnancy, thyroid hormones, micronutrient supplementation

## Abstract

The thyroid gland plays a crucial role in regulating metabolism and supporting development through the production of the hormones T4 and T3. These hormones are essential during childhood for nervous system myelination, physical growth, puberty, skeletal and dental maturation, and overall metabolic balance. In early infancy, when the hypothalamic–pituitary–thyroid axis is still immature, thyroid dysfunction can result in a range of long-term complications. The metabolism and action of thyroid hormones depend not only on iodine but also on other vital micronutrients, particularly selenium (Se). This narrative review aims to comprehensively examine the role of selenium in maintaining thyroid health from fetal life through adolescence. Selenium is a key micronutrient involved in thyroid development, hormone synthesis, antioxidant defense, and immune regulation, especially during pregnancy and childhood. Inadequate selenium levels may contribute to the onset, progression, and clinical management of various thyroid disorders, particularly hypothyroidism and autoimmune thyroid diseases. Although scientific evidence supports selenium’s critical functions in hormone metabolism and antioxidant protection, public awareness and monitoring of selenium intake remain insufficient. Beyond the need for further research, there is an urgent call for integrated public health strategies, ranging from sustainable, food-based approaches to targeted clinical screening and educational programs. Promoting awareness of selenium’s importance and incorporating selenium status into maternal and pediatric care protocols could play a significant role in preventing deficiencies and supporting long-term endocrine and neurodevelopmental health.

## 1. Introduction

The thyroid gland plays a fundamental role in regulating metabolic and developmental processes by producing thyroid hormones, primarily thyroxine (T4) and triiodothyronine (T3) [1]. These hormones are critical throughout childhood, influencing nervous system myelination, somatic growth, pubertal development, skeletal and dental maturation, metabolic homeostasis, and overall organ function [1]. Particularly in early infancy, when the hypothalamic–pituitary–thyroid axis is still immature, thyroid dysfunction can lead to irreversible neurodevelopmental damage and growth delays, emphasizing the need for adequate thyroid function during this highly vulnerable stage [2].

The metabolism and function of these hormones depend not only on iodine but also on other micronutrients, especially selenium (Se). Although elemental Se is not biologically active on its own, it exerts its biological functions through its incorporation as selenocysteine and selenomethionine into twenty-five selenoproteins encoded by the human genome, many of which are directly involved in thyroid hormone synthesis, conversion, and protection against oxidative stress [3,4,5].

Among the most important selenium-dependent enzymes are the iodothyronine deiodinases (DIO1, DIO2, and DIO3), which are responsible for the activation and inactivation of thyroid hormones [6,7]. Specifically, DIO1 and DIO2 catalyze the conversion of the relatively inactive T4 into the biologically active T3. Furthermore, selenoproteins such as glutathione peroxidases and thioredoxin reductases neutralize hydrogen peroxide and other reactive oxygen species (ROS) generated during hormone synthesis, thereby preventing oxidative damage to thyroid cells [6,7].

Se supplementation has shown potential benefits at various stages of pediatric development, although its long-term clinical efficacy remains uncertain. During pregnancy, adequate maternal Se intake has been associated with a lower risk of postpartum thyroiditis and stabilization of thyroid autoantibodies, suggesting a possible role in fetal thyroid [8,9,10]. In infancy and early childhood, although data are still limited, maintaining optimal selenium status may support thyroid hormone metabolism and antioxidant defense mechanisms, both crucial for proper neurodevelopment.

As children grow, particularly during late childhood and adolescence, emerging evidence highlights the modulatory role of Se in thyroid autoimmunity, especially in Hashimoto’s thyroiditis (HT), a condition frequently characterized by elevated thyroid autoantibodies and, in some cases, selenium insufficiency [10,11,12]. Se contributes to the activity of antioxidant enzymes, potentially reducing thyroidal inflammation and oxidative stress, both implicated in the pathogenesis of autoimmune thyroid disease.

Some research indicates that selenium supplementation could positively influence thyroid autoantibodies and thyroid function by enhancing antioxidant defenses and promoting the activation of regulatory T cells [4,13]. However, findings in children remain more variable than in adults [14,15], underscoring the importance of age-specific responses and supporting the need for personalized supplementation strategies, particularly in selenium-deficient pediatric patients or those with established thyroid dysfunction [16,17,18,19].

This review aims to comprehensively explore the role of Se in maintaining thyroid health, from fetal life through adolescence. By synthesizing current evidence, we seek to clarify the physiological relevance of Se in thyroid development, hormone synthesis, antioxidant defense, and immune regulation. Particular attention will be given to the consequences of inadequate Se levels during critical developmental windows, the implications for clinical practice, and public health strategies aimed at ensuring adequate Se intake during early life stages.

## 2. Methods

A narrative review was conducted to explore the role of Se during the stages of organ development and functional maturation, with particular attention to its potential impact on the onset of thyroid diseases, especially in pediatric populations. The search, covering publications from 2005 to 2025, was performed using PubMed and Scopus databases. The keywords used, individually or in combination, included the following: selenium, thyroid function, thyroid disorders, pediatric, children, pregnancy, fetal development, autoimmune thyroiditis, oxidative stress, thyroid hormones, trace elements, and micronutrient supplementation.

Inclusion criteria comprised original research articles, clinical trials, meta-analyses, and reviews published in English in the last 10 years. Studies with a primary focus on pediatric age groups were considered; however, articles involving adult populations were also included if deemed relevant to the topic or useful in understanding underlying mechanisms.

Exclusion criteria included editorials, commentaries, letters, case reports, and all articles not published in English.

The initial search included 3875 records. After removing duplicates and screening for relevance, 581 articles remained. Of these, 103 full-text articles were assessed, and 68 were deemed suitable for in-depth analysis. Reference lists of selected studies were also examined to identify additional relevant sources, including foundational works and studies involving animal models where pertinent to mechanistic understanding.

**Figure 1:**
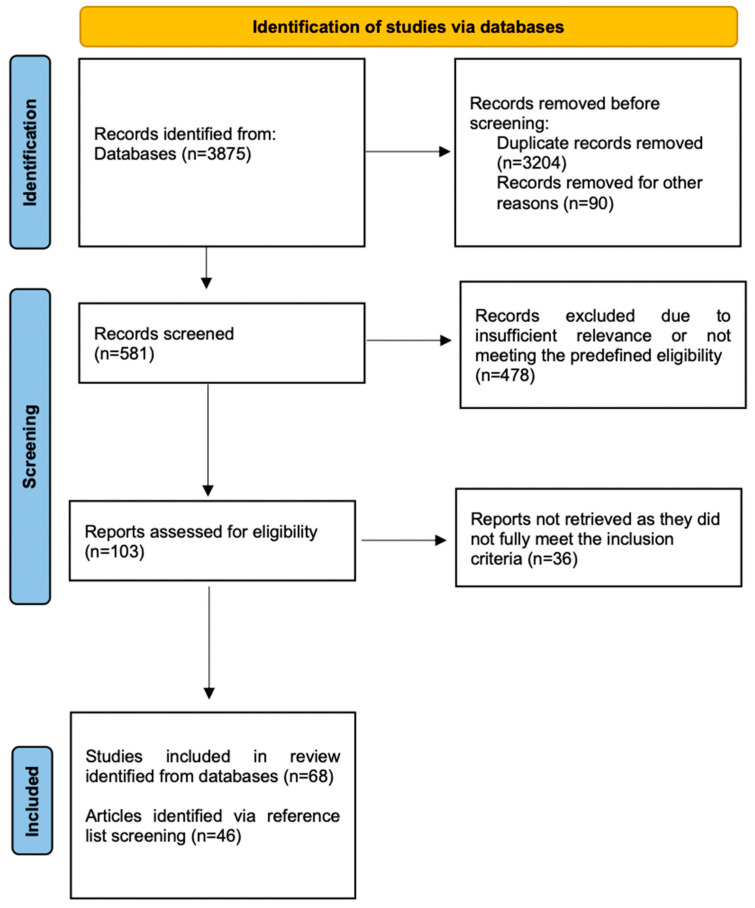
Flowchart illustrating the selection process.

A flowchart summarizing the selection process is provided in Figure 1.

## 3. Thyroid Health and Selenium

### 3.1. Thyroid Development and Selenium

Thyroid development begins very early during embryogenesis and plays a crucial role in regulating metabolism, growth, and the maturation of the central nervous system. The thyroid gland is derived from the endoderm of the anterior pharyngeal floor, where around the third week of human embryonic development a cell thickening forms between the first and second pharyngeal arches [20].

This group of cells begins to express specific genes such as NKX2.1, PAX8, FOXE1, and HHEX, which drive the differentiation of the thyroid primordium [20,21,22]. In animal models, such as the mouse and zebrafish, these genes show activation patterns very similar to those in humans, which has enabled a better understanding of the order in which they act and how important they are for the survival and multiplication of the cells that will give rise to the thyroid [21,23,24].

The thyroid primordium invaginates and forms the thyroid diverticulum, which migrates caudally along the midline to become definitively located in front of the trachea. Errors in this migration can result in congenital thyroid dysgenesis, such as ectopic thyroid (often sublingual), persistent thyroglossal duct, midline cysts, hemiagenesis, or complete agenesis [20,21], frequently associated with mutations in these same regulatory genes [22,25].

Subsequently, the thyroid acquires a follicular structure, within which thyrocytes produce thyroglobulin and begin to concentrate iodine, although autonomous hormone synthesis does not become active until the tenth to twelfth gestational week [20].

At this stage, endocrine function is partly supported by maternal thyroid hormones, which cross the placenta and are crucial for fetal neurocognitive development.

Studies in zebrafish showed that thyroid differentiation is influenced by external signals such as FGF and BMP secreted by the cardiac mesoderm, suggesting an early tissue-specific interaction that was later confirmed in mammals [23].

After birth, the cold extra-uterine environment stimulates an acute increase in TSH, which induces a rapid activation of the neonatal thyroid and a spike in T3 and T4 necessary for metabolic adaptation [26].

During infancy, thyroid hormones are crucial for bone growth, myelination, and regulation of energy metabolism. Their deficiency can result in congenital hypothyroidism with permanent neurological outcomes if not treated early [26,27].

In childhood, the thyroid gland undergoes dynamic modulation, with more active cell proliferation than in adulthood, as demonstrated by histological studies showing a high percentage of cells in mitosis in childhood thyroid tissue. During adolescence, the thyroid gland participates in pubertal development through interaction with the hypothalamic–pituitary–gonadal axis, and in females in particular may show a physiological volumetric increase [27]; an increased incidence of autoimmune diseases such as HT is also observed at this stage, suggesting an influence of sex steroids on thyroid immune function [28]. In adulthood, thyroid physiology remains critical for the regulation of basal metabolism, thermogenesis, and mood [29,30].

Recently, an advanced three-dimensional model of human thyroid development was obtained by culturing organoids derived from fetal thyroid tissue (hFTOs). This system made it possible to observe in vitro key events of follicular maturation, including T4 production and activation of the cAMP-mediated pathway, faithfully mimicking the stages of fetal thyroid development between 12 and 16 weeks of gestation. The study showed that activation of the cAMP signal by forskolin promotes follicle maturation and the expression of genes such as TPO, TG, SLC5A5, and DIO2, suggesting that this pathway may be central to the acquisition of endocrine function. Organoids have been shown to maintain the molecular characteristics of the fetal thyroid and produce hormones in response to TSH, offering a useful model for both the study of normal development and the pathogenesis of congenital defects such as thyroid dysgenesis [26].

Finally, external environmental factors such as iodine deficiency, exposure to goitrogenic substances, nutritional disorders, or viral infections during pregnancy can interfere with proper thyroid development, and this has been demonstrated both in epidemiological studies and in animal models of maternal exposure [24,31].

The combined contribution of in vitro studies, animal models, and clinical observations has allowed us to understand the precise sequence of events that drive embryogenesis and postnatal maturation of the human thyroid gland, with fundamental implications for early diagnosis and prevention of congenital thyroid disorders [26].

Se appears to play a crucial role in the development of the thyroid gland; in fact, during pregnancy, an adequate intake of Se is crucial to ensure the normal proliferation and migration of trophoblasts (cells crucial for placental implantation and development), reducing mitochondrial oxidative stress. Experimental studies also suggest a potential use of selenium in reproductive medicine to improve trophoblast survival and invasion in cases of infertility [32].

A recent review by Minnetti et al. [33] found that pregnancy results in an increased demand for Se to support both maternal and fetal thyroid function. At the same time, however, maternal Se levels often decrease during pregnancy due to hemodilution, increased renal excretion, and increased demand for fetal growth [34]. Se deficiency in pregnancy has been associated with fetal thyroid hypoplasia, reduced follicle number and size, increased oxidative stress, decreased thyroid antioxidant capacity, and, consequently, alterations in thyroid hormone production [34,35,36].

Two different forms of congenital hypothyroidism (myxedematous vs. neurological “cretinism”) have been characterized and associated with combined iodine and Se deficiency. Myxedematous hypothyroidism may occur if the deficiency occurs late in pregnancy, whereas neurological cretinism develops as a result of early deficiency during the first trimester. Both forms result in severe and irreversible mental and physical deficits [3].

Data from observational studies on pregnant women show that Se deficiency during gestation is common, with plasma levels decreasing as pregnancy progresses. Wesolowska et al. emphasize that Se intake during pregnancy (~70 µg/day) is crucial not only for optimal maternal and fetal thyroid function, but also because Se deficiency (<70 µg/L) may also increase the risk of pre-eclampsia, gestational diabetes, premature delivery, and poor fetal neurodevelopment [34].

The thyroid gland differs from most organs in its ability to selectively retain and accumulate Se to meet its high metabolic demands. However, under conditions of increased Se requirements, such as pregnancy, the maternal thyroid becomes more sensitive to the availability of Se transported by SELENOP [3].

In a cohort study conducted in Bangladesh on 750 pregnant women, the concentration of Se in red blood cells (Ery-Se) was measured at week 30 of gestation. It was found that a 0.50 μg/g Hb increase in Ery-Se was associated with an improvement in language comprehension (+3.7 points) and psychomotor development in girls (+12 points) [37].

The Spanish INMA project also observed an “inverted U-shaped” relationship between maternal Se concentration and child neuropsychological development: too low or too high Se values proved unfavorable [38].

It should be noted that, despite these data, supplementation in pregnancy is still not recommended by major international guidelines, such as those of the American Thyroid Association, due to still insufficient evidence and the risk of toxicity due to the mineral’s narrow therapeutic range [39].

### 3.2. Thyroid Function and Selenium

The primary source of Se is the dietary intake, although the amount of the mineral varies depending on the region’s geographical characteristics, such as the concentration of Se in the soil and ocean, and climate [40]. Se can be found in both inorganic forms (selenate [Me_2_SeO_4_] and selenite [Me_2_SeO_3_]) and organic forms (selenomethionine [SeMet] and seleniocysteine [SeCys] or others such as methylselenocysteine, selenocystathionine, and proteins containing these amino acids) [41]. Depending on its chemical form, Se is absorbed through the intestine in different ways: selenate is imported by a sodium/selenate OH–antiporter; selenite is absorbed by simple diffusion; and Se-containing amino acid absorption is based on Na-dependent amino acid transport [42].

The only Se-containing proteins that carry out structural or enzymatic functions for cellular metabolism and homeostasis are selenoproteins, which are defined by the presence of at least one selenocysteine residue incorporated into their structure. These proteins play the key role in the beneficial effects of Se on human health due to the redox-active properties of SeCys at their active site [43]. SeCys, which is regarded as the twenty-first amino acid, is a functional analogue of cysteine that has a Se atom in place of the sulfur atom. Biosynthesis of selenoproteins is a complicated process. SeCys is synthesized on a particular tRNA (selenocysteinyl tRNA^SeRSeC^) that has the UCA anticodon complementary to the UGA stop codon [42]. Selenoprotein is exclusively encoded by the UGA stop codon in the presence of the SeCys insertion sequence (SECIS) element and protein elements such as the SECIS binding protein 2 (SBP2) [44]. Selenoproteome is encoded by 25 genes, but not all the selenoproteins functions are known. Selenoproteins are most abundant in the thyroid, which is also the organ with the highest Se concentration per tissue unit [3].

The thyroid gland plays a crucial role in growth and development, homeostasis, and cardiovascular, neurological, and reproductive functions. Thyroid hormones are essential for metabolic processes such as thermogenesis, establishing basal metabolic rate, and energy expenditure. Thyrotropin-releasing hormone (TRH), thyroid-stimulating hormone (TSH), triiodothyronine (T3), and thyroxine (T4) mediate thyroid function, which is controlled by the hypothalamic–pituitary–thyroid axis [45].

Selenoproteins play a number of biological roles in the thyroid, including catalyzing enzymatic redox reactions, controlling the metabolism of thyroid hormones, and preventing inflammation and oxidative DNA damage brought on by free radicals produced during the thyroid hormone production process, such as hydrogen peroxide (H_2_O_2_) and lipid hydroperoxide.

The three main selenoprotein groups that are intimately associated with thyroid metabolism and healthy function are glutathione peroxidase (GPX), iodothyronine deiodinase (DIO), and thioredoxin reductase (TXNRD) [41].

Hydrogen peroxide (H_2_O_2_) signaling, hydroperoxide degradation catalysis, and cellular redox homeostasis maintenance are among the biological functions of the GPXs, which are antioxidant enzymes. The thyroid contains the proteins GPX1, GPX3, and GPX4, which are principally in charge of protecting the glands from oxidative stress by turning excess oxygen free radicals (H_2_O_2_) that are involved in the production of thyroid hormones into water [46].

Deiodinases are integral membrane selenoproteins that range in size from 29 to 33 kDa. They are composed of a single transmembrane domain and can form dimeric structures. There are three DIO proteins, and their function in physiological settings is to preserve the body’s thyroid hormone homeostasis and activity. SeCys is found in the *N*- terminal portion of these enzymes and is essential for the nucleophilic attack that takes place during the deiodination process. D2 also has a second SeCys in the *C*-terminal part of the protein, but its function is still mostly unknown. Both DIO1 and DIO2 are in charge of using outer ring deiodination to convert the inactive precursor, thyroxine (T4), to the active form of triiodothyronine (T3). DIO3 inactivates thyroid hormones by using inner ring deiodination to convert T4 to reverse T3 (rT3). D2 is located at the endoplasmic reticulum membrane, while D1 and D3 are at the plasma membrane level. D2 and D3 are engaged in the local control of intracellular T3 concentration, and the primary determinant of biological response depends on D2 and D3 activity in the target tissues, while D1 is responsible for regulating thyroid hormone levels in the bloodstream. A feedback loop involving T3 and thyroid-stimulating hormone (TSH) controls the expression of DIOs [47].

The redox signaling system, transcription, immunomodulation, and cell growth factors are all regulated by the TXNRDs selenoenzymes, which have a broad variety of disulfide-containing substrates besides thioredoxin (Trx). The oxidoreductase activity of these selenoproteins has a crucial role in regulating the DNA synthesis process, the transcription of numerous inflammatory genes, and the signaling pathways for apoptosis. The TXNRD system protects the host against oxidative and inflammatory stress and damage and prevents their associated diseases [48,49,50].

The uptake mechanisms underlying thyroid follicles’ deposits of Se are unknown. While Se pools in the liver, muscles, and serum are rapidly depleted, the distribution of Se to the thyroid is preferentially favored in the condition of Se deficiency.

SEPP1 is a protein that mediates the Se supply to some organs, such as the kidney and testis. The fact that SEPP1 deletion had no effect on thyroid function suggests that the thyroid gland may be able to effectively absorb, store, and recycle Se even in the absence of this protein. Since liver-secreted SEPP1 is the primary factor influencing plasma Se levels, thyroidal Se content is usually not represented by serum Se levels. Therefore, indicators of Se bioavailability and cellular activity specific to thyroid tissue have not yet been found [47,51,52].

Even while the thyroid can retain and use Se effectively, a persistent Se deficit negatively affects selenoprotein production and, in turn, thyroid hormone metabolism, which can result in thyroid diseases [40].

**Figure 2:**
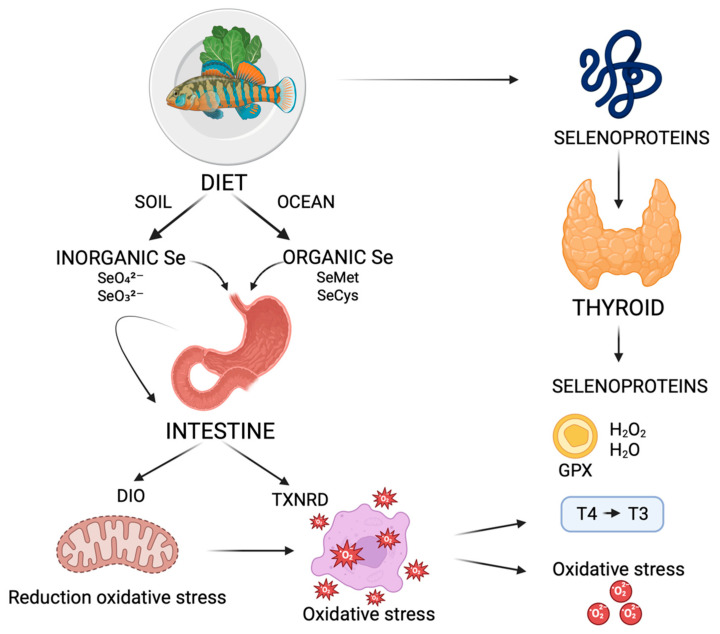
Thyroid function and selenium (created with © 2025 BioRender, 15 June 2025). Se: selenium; SeMet: selenomethionine; SeCys: seleniocysteine; GPX: glutathione peroxidase; DIO: iodothyronine deiodinase; H_2_O_2_: hydrogen peroxide; H_2_O: water; TXNRD: thioredoxin reductase; T3: triiodothyronine; and T4: thyroxine.

Figure 2 schematically illustrates the main steps in the interaction between selenium and the thyroid.

## 4. Selenium Intake During Developmental Stages

Se is an essential trace element required for numerous biological processes, including antioxidant defense, thyroid hormone metabolism, immune function, and neurodevelopment. In infants and pre-school children (ages 0–6 years), Se plays a critical role in growth, cognitive development, and infection resistance. Se requirements in children vary with age and developmental needs.

The European Food Safety Authority (EFSA) and the World Health Organization (WHO) have proposed different reference values for selenium intake in children, reflecting variation in methodology, target populations, and health priorities. Specifically, as reported in Table 1, the EFSA sets adequate intake (AI) levels based on biochemical markers such as selenoprotein P and glutathione peroxidase activity [53], and the WHO provides Recommended Nutrient Intakes (RNI) that reflect the minimum intake to prevent clinical deficiency across diverse global settings [54].

According to reference Intake Levels of Nutrients and Energy for the Italian Population (LARN), the population reference intake (PRI) for Se is 15 µg/day for children aged 1–3 years, increasing progressively to 25 µg/day (4–6 years), 40 µg/day (7–10 years), 50 µg/day (11–14 years), and reaching adult levels of 55 µg/day in adolescents aged 15–17 years. These values are established to ensure adequacy in physiological functions without reaching potentially harmful excesses [55]. Although LARN does not prescribe specific food portions based on Se content alone, the standard portion sizes for Se-rich foods, such as fish (150 g), eggs (50 g), and cereals (80 g for pasta and rice), are structured to help meet these intake goals when consumed according to national dietary guidelines [55].

Both EFSA and LARN emphasize the importance of dietary sources over supplementation and call for more pediatric-specific data to refine selenium intake recommendations and establish reliable biomarkers of adequacy [56].

Despite its importance, both Se deficiency and overexposure remain global health concerns due to the narrow range between nutritional requirement and toxicity [57].

Understanding age-specific Se requirements is essential to ensure optimal thyroid health throughout pediatric life [10]. Its importance is particularly pronounced during pediatric growth, as adequate intake supports normal neurological and endocrine development [58]. The body’s Se requirement differs significantly from infancy to adolescence, influenced by factors such as dietary habits, geographical location, and underlying health conditions [59]. Understanding these requirements, along with the best food sources and evidence-based supplementation strategies, is crucial for ensuring optimal thyroid health in children.

Maternal selenium status during pregnancy is increasingly recognized as a key factor in fetal development. Adequate selenium levels help maintain oxidative balance, support endocrine function, and ensure normal organogenesis. Conversely, selenium deficiency has been associated with impaired intrauterine growth, heightened oxidative stress, and an increased risk of metabolic disorders later in life. Evidence from both human and experimental studies suggests that sufficient maternal selenium intake promotes healthy pregnancy outcomes and may offer early protection against oxidative stress-related conditions in the offspring. These findings highlight the importance of maintaining optimal selenium levels not only after birth but also throughout the earliest stages of development [60]. Maternal serum Se levels below 70 μg/L have been associated with increased risks of low birth weight and congenital hypothyroidism. In a prospective cohort, 8% of women were selenium-deficient and 45% had suboptimal levels (70–99 μg/L). Deficiency correlated with higher rates of thyroid autoimmunity and a significantly increased risk of severe preeclampsia, particularly in twin pregnancies [61].

Human breast milk is the primary Se source during early infancy, and its Se concentration reflects maternal intake. Infant formulas are regulated but may lead to excess Se intake if used exclusively at high volumes. After weaning, key dietary Se sources include fish, meat, eggs, cereals, and dairy products [62]. In breastfed infants, Se status reflects maternal dietary intake, while in formula-fed infants it depends on prenatal stores and formula fortification. Although organically bound forms, particularly Se-enriched yeast, show superior bioavailability, most infant formulas still use inorganic Se. Further research is warranted to evaluate the efficacy and safety of Se-enriched yeast in infant nutrition [63].

Selenium fortification of infant formulas plays a critical role in bridging the gap in selenium status between breast-fed and formula-fed infants. While both organic and inorganic forms are used, evidence suggests that organically bound selenium, such as selenomethionine or selenium-enriched yeast, offers superior bioavailability and tissue retention compared to inorganic forms like selenite or selenate [62].

In an Italian cohort, mean Se concentration in breast milk was 12.1 ng/g, which is adequate for most exclusively breastfed infants, though slightly below international benchmarks. Maternal egg intake during pregnancy and fish consumption during lactation were modestly associated with higher milk selenium levels, underscoring the greater influence of maternal diet during lactation [64]. Despite the role of maternal diet in modulating Se levels in breast milk, specific populations, such as preterm infants, remain particularly vulnerable to selenium deficiency due to physiological and developmental factors. However, preterm infants are at greater risk of Se deficiency due to reduced transplacental transfer and limited body stores at birth [65]. During the transition to solid foods, Se intake in children increasingly reflects dietary choices, with animal products serving as primary sources. Adequate intake is essential for antioxidant defense, thyroid function, and immune development. Regional variability in soil Se and dietary habits highlights the need for balanced intake to prevent both deficiency and excess [56].

Children on plant-based diets may have suboptimal Se intake due to limited reliable sources. Brazil nut products have demonstrated efficacy in improving Se status and may serve as a viable alternative to supplementation in low-intake populations [66]. European data highlight significant regional differences in Se intake among children [67]. Northern European countries, such as Sweden and Finland, generally report higher selenium levels due to naturally selenium-rich soil, with children consuming around 45–60 μg/day [68]. Research from the SENDO project in Spain found that children with diets high in processed foods had significantly lower Se levels compared to those consuming more whole foods [69].

## 5. Clinical Nutrition Insights

In general, epidemiological research on the global burden of Se deficiency is lacking, and studies addressing this topic are very heterogeneous and limited.

Currently, Se deficiency affects an estimated 500 million to 1 billion individuals worldwide, predominantly due to inadequate Se intake from dietary sources [59]. Children and adolescents can be at higher risk of Se deficiency due to environmental factors (e.g., specific features of the soil), clinical determinants, and dietary patterns [56].

Notably, Se deficiency is a relevant public health issue in some regions of China, where soil is poor in Se and Keshan disease is endemic [56]. Considering Africa, Se is among the minerals for which the risk of developing a deficiency due to inadequate dietary intake is higher [70].

In the European context, available data on Se deficiency among pediatric and adolescent populations are scarce and largely outdated [71,72]. A report [71] summarizes data about children and adolescents aged 4–18 years from 16 European countries, referring to a time span between 2000 and 2008. Age-specific values of Se intake were adequate according to D-A-CH (Germany, Austria, Switzerland) 2000 recommendation [71]. Mensink et al. [72] led a systematic review aimed at mapping the prevalence of low micronutrient intakes in Europe. The percentage of children aged 1–3 years with Se intakes below the lower reference nutrient intake (LRNI) was 1% in the UK and 6% in Belgium. Regarding children aged 4–10 years, individuals with Se intakes below the LRNI were under-represented (1.5%) and the highest percentages with insufficient Se intake were observed in Belgium and specifically among girls. Lastly, percentages of adolescents aged 11–17 years with Se intake below the LRNI ranged from 32% to 60% among girls and from 18% to 27% among boys [72].

Although Se deficiency is rare in high-income countries, there are some pediatric populations at higher risk of developing Se deficiency because of diseases involving diet restrictions.

For example, phenylketonuria (PKU) dietary treatment consists of a diet low in natural proteins and Se-rich products such as whole grains, fish, meat, and eggs [56,73]. Consequently, lower Se serum levels can be more frequent among people affected by PKU than in the healthy population [73,74]. Dietary patterns of these patients often do not meet the recommended dietary allowance of Se [73,74]. Thus, according to evidence, diet therapy of these patients should also eventually include Se supplementation in order to support an adequate nutritional status [56,75].

Moreover, pediatric patients affected by intestinal bowel diseases (IBDs) are at higher risk of micronutrient-related malnutrition, and different factors, such as chronic inflammation, intestinal malabsorption, and restrictive dietary patterns, may contribute to these deficiencies [76,77]. Specifically, a reduced Se intake and the involvement of the small intestine (the main site of Se absorption) by disease progression could contribute to Se deficiency in these patients [76]. Nevertheless, routine measurements and supplementation of Se are not suggested in children with IBD [78].

Further, food choices can also affect the risk of Se deficiency. Indeed, a cross-sectional study found a significant inverse association between children’s consumption of ultra-processed foods (UPF) and the intake of Se and other micronutrients [69]. In another study [79], authors found out that the percentage of total energy intake derived from processed food consumption was significantly associated with the prevalence of inadequate Se intake. Indeed, UPF-rich diets provide lower contributions of proteins, fiber, vitamins, and minerals [80]. Besides, UPF consumption in childhood may increase BMI and percentage body fat and determine a higher risk of overweight and obesity [81,82]. A cross-sectional study [83] found out that Se serum and urine levels were significantly lower in children with obesity than in healthy controls. Further, the study showed that Se serum level was a significant independent factor influencing the risk of childhood obesity [83].

Lastly, preterm infants on parenteral nutrition (PN) therapy are at higher risk of Se deficiency [84]. According to recommendations [85], PN therapy should provide a dose of Se equal to 2–3 mg kg^−1^ day^−1^, but some reports affirm that preterm infants’ requirements could be higher [86]. Indeed, in a study on infants on PN therapy with an initiation dose of Se equal to 2–3 mg kg^−1^ day^−1^, the prevalence of Se deficiency was 80%. Notably, this prevalence was higher in extremely low birthweight (ELBW) and very low birthweight (VLBW) participants. The study found that the odds of Se deficiency in ELBW and VLBW infants were higher compared to normal birth-weight participants [84].

Early identification of pediatric populations at higher risk of selenium deficiency is crucial. This includes infants receiving parenteral nutrition, children with diseases requiring restrictive diets, and those following unhealthy lifestyles. Further research is especially needed to develop clear dietary guidelines for micronutrient supplementation in children with obesity, who are more vulnerable to such deficiencies [87]. Although some evidence exists regarding selenium supplementation in infants on parenteral nutrition, further investigation is warranted due to their high-risk status.

## 6. Evidence on the Role of Selenium in Thyroid Disorders

Numerous studies in adults have explored the role of Se in thyroid disorders [4], providing a foundation for understanding its relevance in pediatric populations.

Bano et al. recently reported that thyroid dysfunction, including abnormalities in thyroid hormone synthesis and the emergence of autoimmune thyroid conditions such as Graves’ disease and HT, has been linked to Se deficiency [4]. Their review further explains that selenium deficiency contributes to the pathogenesis of autoimmune thyroid diseases through several mechanisms. First, selenium is essential for the function of antioxidant selenoproteins, such as glutathione peroxidases and thioredoxin reductases, which neutralize reactive oxygen species generated during thyroid hormone synthesis. In conditions of selenium deficiency, reduced antioxidant capacity leads to increased oxidative stress and damage to thyroid follicular cells, promoting the release of autoantigens and triggering autoimmune responses. Second, selenium is required for optimal activity of iodothyronine deiodinases, enzymes that regulate thyroid hormone metabolism by converting inactive T4 to active T3. Impaired deiodinase function due to insufficient selenium may disrupt thyroid hormone homeostasis and contribute to thyroid dysfunction. Finally, selenium plays a role in modulating immune responses by influencing regulatory T cells (Tregs), which help maintain self-tolerance. A lack of selenium may reduce Treg activity and favor a pro-inflammatory environment, further supporting the development and progression of autoimmune thyroid disorders [4].

The study by Rostami et al. highlighted that adults with HT and Se deficiency had elevated TPO-Ab levels, increased thyroid volume, and higher TSH, reinforcing Se’s antioxidative and immunomodulatory relevance [88].

In detail, the cross-sectional analysis conducted on newly diagnosed HT patients showed that those with serum selenium concentrations below 0.85 µmol/L had significantly higher TPO-Ab titers, larger thyroid glands (median volume ~14.4 mL vs. 9.4 mL in Se-sufficient), and elevated TSH compared to Se-replete individuals [88]. Mechanistically, their findings align with the pathophysiological framework outlined by Bano et al. [4].

These data collectively emphasize that selenium deficiency in HT establishes a self-perpetuating cycle of oxidative stress, autoimmunity, structural thyroid alterations, and hormonal dysregulation. Ensuring adequate selenium status may thus represent an important strategy to break this cycle and mitigate disease progression.

A meta-analysis by Wichman et al. demonstrated a significant reduction in TPO-Ab following Se supplementation, especially among patients concurrently treated with levothyroxine (L-T4) [89,90]. In this analysis, the majority of included randomized controlled trials employed a selenium supplementation dose of 200 µg/day, predominantly in the form of selenomethionine, administered over a period ranging from 3 to 12 months. The pooled results indicated that in L-T4-treated patients, selenium supplementation resulted in a mean decrease in TPO-Ab levels of approximately 271 IU/mL at 3 months, with reductions persisting at 6 and 12 months. In selenium-supplemented patients not receiving L-T4 therapy, the antibody reduction was more pronounced at 3 months (mean decrease ≈ 512 IU/mL), but this effect was attenuated at subsequent follow-up points [89].

Despite these biochemical improvements, subsequent systematic reviews have emphasized that the clinical impact of selenium supplementation remains modest, with only slight improvements observed in thyroid ultrasound echogenicity and patient-reported quality of life parameters [91]. Notably, while selenium supplementation at 200 µg/day can effectively reduce autoantibody titers, its translation into meaningful clinical benefit requires further investigation. More recent meta-analyses have reinforced Se’s potential to decrease TPO-Ab and TSH levels in euthyroid and subclinical hypothyroid patients not receiving thyroid hormone replacement therapy, suggesting that benefits may be more evident in early or untreated disease stages [17,92].

The meta-analysis by Huwiler et al. (2024) [17] analyzed randomized trials where selenium was supplemented at doses between 100 and 200 µg/day, primarily as selenomethionine or sodium selenite, over 3 to 12 months. Significant reductions in TPO-Ab and TSH levels were observed, particularly in patients not on levothyroxine therapy.

Similarly, Kong et al. (2023) [92] reviewed trials with selenium doses of 80–200 µg/day administered for 3 to 6 months, confirming a significant reduction in TPO-Ab and Tg-Ab levels at 6 months, although not at 3 months.

An observational study supporting this link was conducted by Federige et al. in 2017 on 73 adults affected by Hashimoto’s thyroiditis in Brazil; the authors found significantly lower serum selenoprotein P (SePP) levels in HT patients not on L-T4, with SePP levels correlating inversely with TPO-Ab [93]. Although some trials report improved immunological markers and thyroid imaging, the clinical significance of TPO-Ab reduction and its long-term impact remain uncertain [94].

A lot of research has been conducted on the Se status of pregnant women, looking at how this affects both the thyroid function of the mother and of the fetus. Ambroziak et al., studying 74 pregnant women in Poland, have shown that Se deficiency is common among this population, with Se levels declining throughout gestation. Serum Se and SELENOP concentrations were relatively low in both healthy and AITD pregnant women. However, in this study, Se levels appeared to be independent from thyroid autoantibody levels [95]. In contrast with this evidence, the SERENA study, a multicenter trial performed in Italy on 45 women with thyroiditis, showed that Se at physiological doses during pregnancy can reduce thyroid autoantibody titers and lower the risk of postpartum thyroiditis recurrence without adverse effects. This study supports the potential benefit of Se supplementation at 83 mcg/day during pregnancy and after delivery [96]. Considering the effects on offspring, their risk of Se deficiency has been strongly associated with maternal Se deficiency: a report by Polanska et al. assessed the prevalence of Se deficiency among 683 children (<5 years old) and women of reproductive age in sub-Saharan Africa, showing that children of Se-deficient mothers are up to four times more likely to be deficient themselves [97]. This intergenerational link may contribute to poorer neonatal outcomes, as evidenced by findings that low maternal Se is associated with elevated TSH levels and lower birth weight in newborns [98].

Similarly to adults, in the pediatric population, the recent literature underscores that inadequate Se status may influence the onset, progression, and management of various thyroid disorders, particularly hypothyroidism and AITDs [10,99].

Clinical studies in hypothyroid children from Iran and Ethiopia demonstrate a significant inverse relationship between serum Se levels and thyroxine (T4) concentrations, suggesting a potential disruption in deiodinase activity and peripheral conversion of T4 to triiodothyronine (T3) in Se-deficient states. For instance, Khorasani et al. reported that lower serum Se was associated with lower TSH and higher T4 levels, impacting the levothyroxine dose needed for treatment; these results indicated that Se deficiency could prevent T4 to T3 conversion [100]. Similarly, Gashu et al. observed elevated T4 in severely Se- and iodine-deficient children [101]. Conversely, other studies found no significant difference in Se status between hypothyroid and healthy children, suggesting etiological heterogeneity in Se’s role [102,103].

Autoimmune thyroiditis in childhood, particularly in Down syndrome populations, has been closely linked to Se deficiency. Hisbiyah et al. showed that children with Down syndrome and AITD had significantly lower SePP and GPx3 levels, which negatively correlated with anti-thyroid antibodies (TPO-Ab and TgAb) and positively with thyroid function [12]. This points to a dual role for Se in modulating immune tolerance and protecting thyroid tissue from oxidative stress.

Intervention studies investigating Se supplementation in pediatric HT offer mixed but informative results. In a randomized double-blind trial, supplementation with 200 µg/day of L-selenomethionine for six months led to a significant reduction in anti-thyroglobulin antibodies compared to placebo (Δ −70.9 IU/mL vs. −6.7 IU/mL; *p* = 0.021) [104].

Conversely, other intervention studies reported less consistent findings. Onal et al. found that Se supplementation did not significantly alter antibody titers in euthyroid HT but led to favorable reductions in thyroid volume, indicating morphological improvements even in the absence of biochemical normalization [105]. In their open-label, prospective pilot study, 23 children with euthyroid Hashimoto’s thyroiditis received 50 µg/day of L-selenomethionine for 3 months. Although no significant changes in TPO-Ab or thyroglobulin antibody levels were observed, a significant reduction in thyroid volume was documented in about 35% of participants, suggesting that selenium may exert structural benefits on the thyroid gland independent of its effects on autoantibody production [105].

Other studies suggested that in iodine-deficient regions, addressing concurrent Se deficiency without iodine supplementation heightens the conversion of T4 to T3 in the periphery, further supporting its potential therapeutic role in select contexts and underscoring the need for a balanced micronutrient approach [106].

Despite these findings, not all studies confirm a clear or uniform benefit. Large-scale meta-analyses and randomized controlled trials in adult populations often note modest or no impact of Se supplementation on thyroid function or quality of life, prompting caution in extrapolating such findings to children. For instance, the absence of differences in SePP and GPx levels in well-treated hypothyroid children and the lack of thyroid hormone correlation in congenital hypothyroidism suggest that Se’s influence may be attenuated in the context of optimized therapy or congenital etiologies [107,108].

While evidence highlights Se’s impact on thyroid hormone metabolism, immune modulation, and disease course, the variability across studies underscores the need for individualized assessment of Se status.

Overall, the reviewed evidence suggests that selenium supplementation doses ranging from 80 to 200 µg/day in adults have been most commonly studied, with potential benefits in reducing thyroid autoantibody titers, particularly in early or untreated autoimmune thyroid disease. In children, evidence remains limited and mixed: while a study using 50 µg/day did not show significant changes in antibody levels, a more recent trial with 200 µg/day found a significant reduction in anti-thyroglobulin antibodies. However, safety and optimal dosing still require further confirmation before routine use can be recommended.

Table 2, summarizes the principal studies investigating the role of selenium in thyroid pathophysiology among pregnant women and the pediatric population.

## 7. Effective Strategies to Ensure Adequate Selenium Intake in Children

Nutrition education, prevention strategies, and intervention programs aimed at ensuring adequate Se intake from birth to the different developmental stages have not yet been addressed in the literature. Despite Se’s critical role in child development, few targeted strategies can be significantly improved to promote its adequate intake. Firstly, to ensure adequate intake of Se in children, it is important to optimize maternal Se level during pregnancy, especially during the last trimester when the fetal micronutrient stores are established and maternal Se level tends to decrease [109]. Furthermore, maternal Se deficiency is associated with increased risk of low birth weight in newborns, as shown by correlations between Se concentrations in maternal and umbilical cord blood [110]. Therefore, maintaining proper Se status during pregnancy is vital for neonatal health [10]. Studies suggest a cutoff for maternal serum Se deficiency of 0.90 µmol/L at pregnancy week 18 and 0.78 µmol/L at pregnancy week 36, which should be respected in order to reduce those risks [109]. Moreover, for breastfed infants, maternal Se intake during lactation seems to be enhanced by the consumption of fish, so an adequate intake is recommended (150 g, LARN). For formula-fed infants, the use of formulas with organic Se, less common today than its inorganic sibling, would make it more easily absorbed [55,63,64].

After examining the findings from several studies that support the importance of selenium in infants’ diets, the FDA published a final rule in 2015 that added Se to the list of mandatory nutrients in infant formula and established minimum and maximum levels of selenium in infant formula for both full-term and premature infants. For term infants, findings recommend Se from 2 µg to 15 µg per 100 kcal based on the extremes of the available guidelines (FDA, EFSA, ESPGHAN, Institute of Medicine—IOM). For preterm infants, the available guidelines recommend a minimum of 2 µg kg^−1^ d^−1^ to a maximum of 4.5 µg kg^−1^ d^−1^ (ASCN, ASPEN, ESPGHAN, American Academy of Pediatrics—Committee on Nutrition).

During normal child development, Se level is related to several factors, such as geographical location and its content in the soil [111], but it could also be affected by genetics, age, gender, body weight, supplementation, and diet composition [112]. Mostly in regions with low Se soil concentrations can Se be considered a critical nutrient for vegetarian and vegan children, who often consume less Se than omnivores [67]. However, guidelines for recommended intakes also differ between countries along with eating habits. In those where the consumption of ultra-processed foods (UPF) is high, the deficiency is made more critical, as UPF seems to be associated with an inadequate intake of Se and other micronutrients in a sample of children from the Mediterranean area [69]. In other countries (e.g., Brazil), some children may suffer excessive intake of Se because of the consumption of Brazil nuts, although there are no cases of toxicity to the best of our knowledge [113]. Accordingly, the alignment of portion sizes with nutrient-based recommendations would facilitate practical application in both individual meal planning and public health nutrition strategies.

This also highlights the need for prevention strategies, starting from nutritional education, so that parents and caregivers can ensure the correct intake suggested by the available recommendations, being aware of which food is more or less rich in Se. To date, several studies have confirmed that cereals, meat and dairy products, fish, seafood, milk, and nuts are the primary dietary sources of Se [53,67,112,114]. Therefore, it is important to ensure their adequate intake as part of a balanced diet. Furthermore, it should be noted that, although the guidelines do not recommend a particular form of selenium for selenium fortification, evidence indicates that organically bound selenium formulations (e.g., selenomethionine and selenium-enriched yeast) are better absorbed and retained than inorganic forms [62]. In order to achieve the correct intake, it is crucial for parents and caregivers to comprehend that Se bioavailability and uptake depend on many factors, of which the main ones are its chemical form and other components of the food matrix [56,112]. In particular, Se is efficiently absorbed when present in organic forms, especially in combination with vitamins A, D, and E. Therefore, ensuring an adequate intake of these fat-soluble vitamins, largely dependent on proper nutritional education, is essential to optimize Se bioavailability and support its physiological roles. The bioavailability is determined also by dietary factors such as fat, protein, and heavy metal content [112]. Moreover, although in normally developing children, Se status depends mostly on diet, in children in whom it is crucial to introduce a dietary restriction related to different issues (e.g., food allergies or chronic diseases), it may be more difficult to achieve the recommended intake [69]. As a preventive measure, the sale of UPF products in school vending machines should be restricted in order to address Se deficiency. Additionally, steps should be taken to fortify children’s food with organic Se [62,63] and to update food composition tables to include Se intake [53].

## 8. Limitations

Despite the comprehensive and multidisciplinary approach adopted in this work, several limitations must be acknowledged. Firstly, this study is structured as a narrative review, which inherently carries certain methodological constraints. Unlike systematic reviews or meta-analyses, narrative reviews do not follow a standardized protocol for literature selection and data synthesis, which may introduce selection bias, reduce reproducibility, and limit the generalizability of conclusions.

A further limitation lies in the heterogeneity of the available literature, particularly in the pediatric field. Existing studies differ widely in terms of design, sample size, population characteristics, biomarkers used to assess selenium status, and supplementation protocols. This diversity makes direct comparisons difficult and hampers the ability to draw consistent or broadly applicable conclusions. Another critical issue is the geographical variability in selenium content in soil and food sources, which further complicates the assessment of selenium adequacy. Differences in local diets, cultural practices, and the availability of selenium-rich or fortified foods result in inconsistent intake across regions, which undermines the uniformity of public health strategies and recommendations.

In addition, there is still no universally accepted biomarker of selenium adequacy that reflects thyroid-specific selenium activity. Serum selenium levels, commonly used in studies, may not reliably mirror intracellular availability or the functional status of thyroid selenoproteins. This hampers the accurate monitoring of supplementation efficacy or deficiency in clinical practice.

While some studies suggest a potential benefit of selenium supplementation in autoimmune thyroid disorders in children, the clinical evidence remains limited, inconsistent, and inconclusive. The effectiveness of selenium supplementation appears to depend on multiple variables, including iodine status, baseline selenium levels, disease stage, and the form and dose of selenium used. Moreover, selenium has a narrow therapeutic range, meaning that both deficiency and excess can have harmful effects. This makes broad supplementation strategies in pediatric populations difficult to justify without individualized assessment.

Finally, the translation of current evidence into specific, evidence-based nutritional recommendations for children remains challenging. Additional large-scale prospective studies and well-designed pediatric trials are essential to refine intake guidelines, define safe and effective supplementation protocols, and develop reliable biomarkers for clinical and public health use.

## 9. Future Directions

A starting point would be the use of sustainable food-based strategies to avoid depleting soils, especially in areas at higher risk. Moreover, to our knowledge, no studies have investigated strategies to increase awareness of Se intake in the diet. Accordingly, it is crucial to raise awareness of the importance of Se as a micronutrient deficiency during pregnancy and child development in healthy and at-risk populations. Therefore, secondary and tertiary preventive strategies should be implemented in hospitals that care for pregnant women, including targeted screening and clinical assessments that take Se status into consideration. In addition, these interventions should be supported by primary prevention educational programs that raise awareness about the importance of adequate micronutrient intake, contributing to the overall health of mother–child dyads. Moreover, Se levels should be part of standard newborn screening practices and adopted by pediatricians as a standard check-up to monitor levels to prevent possible deficiency. Furthermore, more research is needed to develop public health strategies that mitigate the risk of micronutrient deficiencies, which raise awareness of the importance of Se both from a preventive point of view and in terms of ensuring a proper Se intake.

## 10. Public Health and Policy Considerations

In most European countries, diet-related diseases pose a serious threat to public health. Malnutrition, including micronutrient deficiencies such as Se, persists in certain areas of the WHO European Region [115].

Food policies have the potential to address micronutrient deficiencies by fostering healthy food environments and employing a multilayered approach involving dietary diversification, food fortification, targeted supplementation, and improved hygiene. However, current strategies often lack robust monitoring indicators to assess short- and long-term impacts and are insufficiently integrated across regional, national, and cross-sectoral initiatives. Rethinking and redesigning food policy goals and strategies thus represents a critical opportunity to tackle micronutrient malnutrition on a broad scale. In 2007, the World Health Organization launched the European Action Plan for Food and Nutrition Policy (2007–2012) [116], in collaboration with Member States, international organizations, NGOs, and public health experts. This plan identified four major health challenges, including the reduction of micronutrient deficiencies through improved nutrition and food safety policies, alongside promotion of physical activity, adequate water intake, and reduced alcohol consumption, in line with WHO and FAO recommendations.

Effective prevention programs should begin during, or even before, pregnancy, laying the foundation for lifelong health. Prevention must take place not only within the family but across all levels of society.

A cohesive national alliance involving authorities, healthcare providers, schools, families, and individuals is essential to align efforts and resources. A coordinated strategy across multiple sectors should ensure access to nutritious foods and promote environments conducive to healthier lifestyles. Key priorities include enhancing fetal nutrition through maternal dietary education and support, implementing pre-school and school-based food and nutrition policies, and reforming trade practices to increase the availability, affordability, and safety of fruits, vegetables, and micronutrient-rich foods, including fortified complementary products.

## 11. Conclusions

Selenium is a key micronutrient in thyroid development and function, particularly during pregnancy and childhood. While scientific evidence supports its role in hormone metabolism and antioxidant protection, awareness and monitoring of selenium intake remain insufficient. The available evidence regarding selenium supplementation at different stages of growth is not entirely complete and does not seem to be uniformly applied. The scientific literature available to date reveals a lack of consistent guidelines, as they are often not adapted to the needs of specific populations, and a clear lack of targeted strategies, such as well-structured educational interventions that focus both on food sources rich in selenium and on its bioavailability. Beyond the need for further research, there is a pressing call for integrated public health strategies: from sustainable food-based approaches to targeted clinical screening and educational programs. To date, there are no comprehensive monitoring and awareness strategies in standard procedures such as pregnancy monitoring and pediatric screenings that would help prevent any comorbidities related to selenium deficiency. Promoting awareness of selenium’s importance and incorporating Se status into maternal and pediatric care protocols could contribute significantly to preventing deficiencies and supporting long-term endocrine and neurodevelopmental health. Further action is needed to strengthen public awareness through education campaigns, food safety guidelines, improved food labeling, and targeted health communication. The healthcare sector bears a significant responsibility in reducing the burden of diet-related diseases, necessitating improvements in prevention, diagnosis, treatment, and surveillance systems at all levels. Given the scale of the global nutrition challenge and the limitations of past approaches, a paradigm shift in food policy is urgently needed. Redefining food policy objectives offers a major opportunity to improve population health and nutrition sustainably. In conclusion, there is considerable opportunity to improve in terms of awareness, prevention strategies, and public education, which need to be further developed.

## Figures and Tables

**Table 1:** Se requirements in children according to the European Food Safety Authority (EFSA) and the World Health Organization (WHO) [53,54].

Age Group	EFSA–AI (µg/day)	WHO–RNI (µg/day)
0–6 months	12.5	6
7–12 months	15	10
1–3 years	15	17
4–6 years	20	22
7–10 years	30	30–40
11–14 years	55	45
14–18 years	65–70	60

**Table 2:** The main studies investigating the role of selenium in thyroid disorders among pregnant women and children.

Author(s)	Study Type	Patients	Location	Main Findings
Wichman et al. (2016) [89]	Meta-analysis	Various	Multiple	↓ TPO-Ab post-Se supplementation, esp. with L-T4
Ambroziak et al. (2017) [95]	Observational	74 pregnant women	Poland	↓ Se during pregnancy; not correlated with autoantibody levels
Mantovani et al. (2019) [96]	Multicenter clinical trial	45 pregnant women with thyroiditis	Italy	83 mcg/day Se ↓ postpartum thyroiditis risk and autoantibodies
Polanska et al. (2017) [97]	Survey/observational	683 children and women	Sub-Saharan Africa	Maternal Se deficiency ↑ child Se deficiency risk 4x
Khorasani et al. (2017) [100]	Clinical study	Hypothyroid children	Iran	↓ Se associated with ↓ TSH, ↑ T4; impacts L-T4 dosing
Gashu et al. (2016) [101]	Clinical study	Children	Ethiopia	Elevated T4 in Se- and iodine-deficient children
Hisbiyah et al. (2023) [12]	Observational	Children with Down syndrome + AITD	Not specified	↓ SePP and GPX3; correlated with ↓ thyroid antibodies, ↑ thyroid function
Onal et al. (2012) [105]	Intervention study	Children with euthyroid HT	Not specified	Se supplementation ↓ thyroid volume, no change in antibody titers
Santos et al. (2018) [106]	Intervention/observational	Children	Iodine-deficient regions	Se alone ↑ T4 → T3 conversion
Santos et al. (2018) [106]	Observational	Children	Not specified	In iodine-deficient regions, Se supplementation alone increases peripheral T4 to T3 conversion
Nourbakhsh et al. (2016) [107]	Observational	Children and adolescents with HT and hypothyroidism	Not specified	No difference in SePP and GPX levels in well-treated patients; limited correlation with thyroid hormones
Blasig et al. (2016) [108]	Observational	Children with congenital hypothyroidism	Not specified	Positive correlation between thyroid hormones and serum copper; Se’s role possibly attenuated

GPX: glutathione peroxidase; HT: Hashimoto’s thyroiditis; L-T4: levothyroxine; SePP: serum selenoprotein P; TPO-Ab: anti-thyroid peroxidase antibodies; ↓: decrease, ↑: increase.
